# Isolation and structure determination of missing fullerenes Gd@C_74_(CF_3_)_*n*_ through *in situ* trifluoromethylation

**DOI:** 10.1098/rsos.181015

**Published:** 2018-09-19

**Authors:** Ayano Nakagawa, Shinobu Aoyagi, Haruka Omachi, Katsuma Ishino, Makiko Nishino, Jeremy Rio, Chris Ewels, Hisanori Shinohara

**Affiliations:** 1 Department of Chemistry, Graduate School of Science, Nagoya University, Nagoya 464-8602, Japan; 2 Research Center for Materials Science, Nagoya University, Nagoya 464-8602, Japan; 3 Institute for Advanced Research, Nagoya University, Nagoya 464-8602, Japan; 4 Department of Information and Basic Science, Nagoya City University, Nagoya 467-8501, Japan; 5 Institut des Materiaux Jean Rouxel (IMN), Université de Nantes, CNRS UMR6502, 2 Rue de la Houssiniere, BP32229, Nantes 44322, France

**Keywords:** metallofullerenes, gadolinium, X-ray crystallography analysis, density functional calculations

## Abstract

Our trifluoromethyl functionalization method enables the dissolution and isolation of missing metallofullerenes of Gd@C_74_(CF_3_)*_n_*. After multi-stage high-performance liquid chromatography purification, Gd@C_74_(CF_3_)_3_ and two regioisomers of Gd@C_74_(CF_3_) are isolated. X-ray crystallographic analysis reveals that all of the isolated metallofullerenes react with CF_3_ groups on pentagons of the *D*_3 h_-symmetry C_74_ cages. Highest occupied molecular orbital-lowest unoccupied molecular orbital gaps of these trifluoromethylated derivatives, estimated by absorption spectra, are in the range 0.71–1.06 eV, consistent with density functional calculations.

## 1. Introduction

Endohedral metallofullerenes M@C_2*m*_ (M = rare earth metal), encapsulating metal atoms in the internal space of spherical carbon structures, have attracted much attention due to their unique properties [1,2]. Of these, gadolinium-encapsulated metallofullerenes have been widely investigated for biomedical applications [3–8]. With such high magnetic moments, Gd@C_2_*_m_* are of interest as novel magnetic resonance imaging (MRI) contrast agents [3–6]. The fully enclosing carbon cage completely prevents leaching of the Gd atoms, resulting in lower toxicity than commercially available metal chelate reagents such as Gd-DTPA. However, only M@C_82_ type metallofullerenes have been used for these applications. Although a lot of small-cage endohedral metallofullerenes M@C_2*m*_ (2*m* = 60, 70, 72 and 74) are obtained in as-synthesized carbon soot, there are few examples of successful isolation [9–15]. These metallofullerenes, so-called missing (or small highest occupied molecular orbital (HOMO)–lowest unoccupied molecular orbital (LUMO) gap) metallofullerenes, are highly reactive and tend to form insoluble polymerized solids in raw soot.

Recently, we developed an *in situ* trifluoromethylation method for the extraction and purification of these missing metallofullerenes [16–19]. CF_3_ groups [20–23], furnished by the thermal pyrolysis of polytetrafluoroethylene (PTFE), were introduced to the outer cages of fullerenes and stabilized reactive missing metallofullerenes during the production simultaneously. A series of yttrium- and lanthanum-encapsulated missing metallofullerenes were isolated by this technique. However structural determination still remains an important open question for fullerene science. We herein report the isolation of CF_3_-functionalized Gd@C_74_ and structural determination by single-crystal X-ray diffraction.

## 2. Method

### 2.1. Synthesis and purification of Gd@C_74_(CF_3_) (I), Gd@C_74_(CF_3_) (II) and Gd@C_74_(CF_3_)_3_

Trifluoromethylated Gd-metallofullerenes were synthesized by the modified arc-discharge method. A cross-sectional view of the DC arc-discharge chamber is illustrated in electronic supplementary material, figure S1, where PTFE rods (40 g) are placed near the discharge area. A graphite rod (100 g) impregnated with Gd (La) (0.8 mol%, Toyo Tanso Co. Ltd) was used as the anode. A pure graphite rod (Toyo Tanso Co. Ltd) was used as the cathode. Arc discharge was performed at a DC current of 500 A in a flowing He atmosphere with a pressure of 7–9 kPa. During arc discharge, because of the high temperature around the arc zone, PTFE was decomposed and evaporated to produce CF_3_ radicals. Normally, 50–70 g of raw soot was obtained per discharge. Gd-metallofullerenes and empty fullerenes were extracted from the raw soot with *o*-xylene.

### 2.2. Separation of trifluoromethylated Gd-metallofullerenes from empty fullerenes by TiCl_4_

To a 500 ml CS_2_ solution of the crude mixture, *ca* 5 ml of TiCl_4_ was added. Metallofullerenes were reacted immediately and insoluble complexes were precipitated out [24–26]. After mixing for 5 min, the precipitate was collected on PTFE membrane filter and washed with 10–20 ml of CS_2_ to separate from the fullerenes in solution. Deionized water was passed through the filter to decompose the complexes of metallofullerene/TiCl_4_, and then washed with acetone to eliminate extra water. Finally, CS_2_ was passed through the filter to collect the desired metallofullerenes as a solution.

### 2.3. Multi-stage high-performance liquid chromatography purification of Gd-metallofullerenes

High-performance liquid chromatography (HPLC) purification was conducted using a JAI (Japan Analytical Industry Co.) recycling preparative HPLC LC-9104HS. Three isomers of Gd@C_74_(CF_3_)*_n_* were isolated from the mixture by the multi-stage HPLC method. Two kinds of columns were used alternatively with toluene eluent for the isolation, i.e. Buckyprep column (20 mm diameter × 250 mm, Nacalai Tesque Inc.) and Buckyprep-M column (20 mm diameter × 250 mm, Nacalai Tesque Inc.). The initial (first-stage) HPLC purification was performed with Buckyprep-M. Gd@C_74_(CF_3_)_3_ was obtained in fraction A and Gd@C_74_(CF_3_) (I) and (II) were obtained in fraction B. The overall separation scheme and the HPLC chromatograms are shown in electronic supplementary material, figures S2–S10.

### 2.4. X-ray crystal structure analysis

Single crystals of Gd@C_74_(CF_3_) (I) and (II) and Gd@C_74_(CF_3_)_3_ were obtained by co-crystallization with Ni(OEP) (OEP = octaethylporphyrin) from solution. The single-crystal X-ray diffraction data were collected at SPring-8 BL02B1 [27]. The crystal structures were determined using SIR [28] and SHELX [29]. The crystallographic data are summarized in electronic supplementary material, table S1, and crystallographic information files (CIF). A theoretical *D*_3h_-symmetry C_74_ rigid-body molecule was used in modelling of the Gd@C_74_(CF_3_)*_n_* molecules showing severe orientation disorder. Although the Gd@C_74_(CF_3_)*_n_* molecules have chiral structures, the space groups of the crystals are centrosymmetric. Therefore, each crystal contains the same number of the chiral isomers. The anisotropic atomic displacement parameters of carbon atoms on disordered C_74_ cages of Gd@C_74_(CF_3_) (I) and (II) were determined by using two parameters of translation and libration motions of the rigid-body molecules [30,31]. The CIF deposition numbers at the Cambridge Crystallographic Data Centre (CCDC) are 1824999 for Gd@C_74_(CF_3_) (I), 1825000 for Gd@C_74_(CF_3_) (II) and 1825001 for Gd@C_74_(CF_3_)_3_.

### 2.5. Density functional calculations

Density functional (DFT) calculations were performed under the local spin density approximation, as implemented in the AIMPRO code [32–34]. Relativistic pseudopotentials were included via the Hartwigsen–Goedecker–Hütter scheme [35]. For C/Gd/F, a basis set containing 38/90/28 independent Gaussian-based functions was used [36]. Calculations were fully spin polarized with spin relaxation. Periodic boundary conditions at the gamma point were used, with cell size large enough to avoid interaction between neighbouring fullerenes. A system-dependent plane wave energy cutoff of 300 Ha (Ha: Hartree energy) was applied with a non-zero electron temperature of *kT* = 0.04 eV for electronic level occupation. Atomic positions were geometrically optimized until the maximum atomic position change in a given iteration dropped below 10^−6^ *a*_0_ (*a*_0_: Bohr radius). The method has been previously successfully applied to study Gd-metallofullerenes [19] and is discussed in more detail in another article [37].

## 3. Results

**Figure 1.**
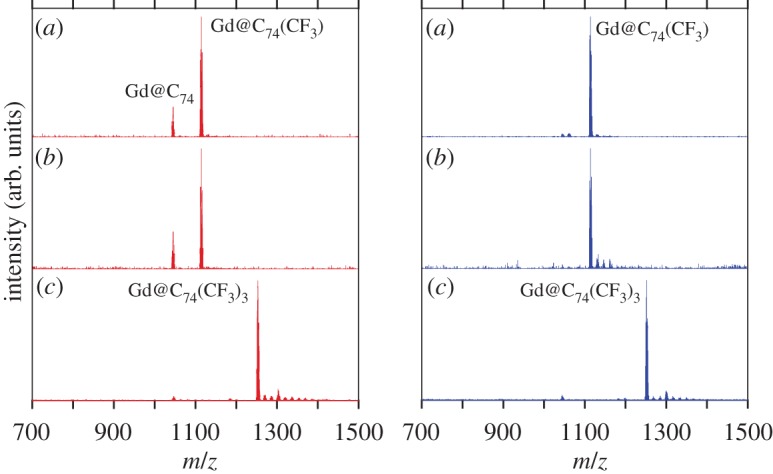
Mass spectra (positive-ion mode in red, negative-ion mode in blue) of (*a*) Gd@C_74_(CF_3_) (I), (*b*) Gd@C_74_(CF_3_) (II) and (*c*) Gd@C_74_(CF_3_)_3_.

**Figure 2.**
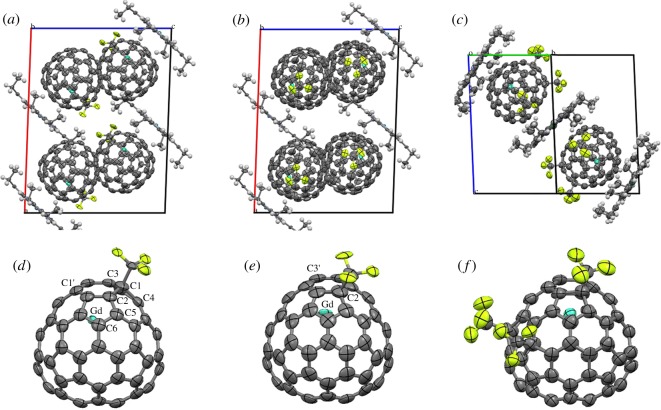
(*a*–*c*) Crystal structures of (*a*) Gd@C_74_(CF_3_) (I), (*b*) Gd@C_74_(CF_3_) (II) and (*c*) Gd@C_74_(CF_3_)_3_, co-crystallized with Ni(OEP). The thermal ellipsoids are drawn at 50% probability level. Hydrogen atoms are drawn as small spheres. Disordered structure and solvent molecules are omitted. View along (*a*,*b*) $\left[1\bar{1}\;0\right]$ or (*c*) $\left[1\bar{1}\;0\right]$. (*d*–*f*) Feasible molecular structures of (*d*) Gd@C_74_(CF_3_) (I), (*e*) Gd@C_74_(CF_3_) (II), and (*f*) Gd@C_74_(CF_3_)_3_ viewed along the three-fold axis of the *D*_3h_-symmetry C_74_ cages.

The purity of the three metallofullerenes obtained by multi-stage HPLC preparation was confirmed by matrix-assisted laser desorption/ionization time-of-flight (MALDI-TOF) mass spectroscopy (figure 1). The mass spectra of the isolated samples show strong isolated peaks of Gd@C_74_(CF_3_) (I and II) and Gd@C_74_(CF_3_)_3_, respectively, confirming that these species are highly purified through the multi-stage HPLC separation. Figure 1*a–c* indicates a peak corresponding to the presence of Gd@C_74_, attributed to the parent fullerene dissociated by laser-induced fragmentation. Close inspection reveals that this peak is enhanced in the positive-ion mass spectra. The preferential detection of Gd@C_74_ in the positive-ion spectra results from the elimination of the electron-withdrawing CF_3_ group. After the dissociation of the CF_3_ moieties, the remaining Gd@C_74_ tends to lose electrons and be detected as a cation [16–19]. Figure 2*a* shows the molecular arrangement of the monoclinic crystal consisting of Gd@C_74_(CF_3_) (I) and Ni(OEP) in a ratio of 1 : 1. The crystal also contains toluene and chloroform solvent molecules. The disordered Gd@C_74_(CF_3_) (I) molecule on the crystallographic mirror plane was modelled by a Gd atom occupying four (two independent) positions and a C_74_(CF_3_) (I) molecule with six (three independent) orientations (electronic supplementary material, figure S11). The complicated disorder can be represented by an overlap of Gd@C_74_(CF_3_) (I) molecules with an ordered structure shown in figure 2*d* with different orientations.

Figure 2*d* shows a feasible molecular structure of Gd@C_74_(CF_3_) (I) derived from the X-ray crystal structure analysis. A CF_3_ group is attached to a carbon atom on a pentagon of the *D*_3h_-symmetry C_74_ cage. The *D*_3h_-symmetry C_74_ cage has six independent carbon atoms on pentagons, which are labelled as C1–C6 in the figure. The structural model with a CF_3_ group attached to the C1 atom gave the best reliable factor in the X-ray crystal structure analysis of Gd@C_74_(CF_3_) (I). The Gd atom locates near the carbon atom labelled as C1′ in figure 2*b*. The C1′ atom is the third nearest to the C1 atom with the CF_3_ group attached. Interestingly, the C1 and C1′ atoms are equivalent in the *D*_3h_-symmetry C_74_ cage with the mirror symmetry. The metal atoms of Gd@C_60_(CF_3_)_5_ (I) and (II), La@C_60_(CF_3_)_5_ (I) and La@C_70_(CF_3_)_3_ also locate near a carbon atom which is the third nearest to a carbon atom with a CF_3_ group attached [17,19]. The interatomic distance between the Gd and C1′ is 2.20 Å in Gd@C_74_(CF_3_) (I), which is slightly shorter than Gd−C distances of 2.35 and 2.38 Å in Gd@C_60_(CF_3_)_5_ (I) and (II), respectively [19]. On the other hand, it is slightly longer than a Gd−C distance of 2.08 Å in Gd@*C*_2*v*_(9)-C_82_ [38].

The molecular arrangement and lattice constants of the Gd@C_74_(CF_3_) (II) crystal are similar to those of the Gd@C_74_(CF_3_) (I) crystal (figure 2*b*). Solvent molecules in the Gd@C_74_(CF_3_) (II) crystal are toluene and carbon disulfide, which are different from those (toluene and chloroform) in the Gd@C_74_(CF_3_) (I) crystal. The disordered Gd@C_74_(CF_3_) (II) molecule on the crystallographic mirror plane was modelled by a Gd atom occupying seven (four independent) positions and a C_74_(CF_3_) (II) molecule with four (two independent) orientations (electronic supplementary material, figure S12). Figure 2*e* shows a feasible molecular structure of Gd@C_74_(CF_3_) (II) derived from the X-ray crystal structure analysis. The structure model with a CF_3_ group attached to the C2 atom gave the best reliable factor in the X-ray crystal structure analysis of Gd@C_74_(CF_3_) (II). The Gd atom in Gd@C_74_(CF_3_) (II) also locates near a carbon atom (C3′) which is the third nearest to a carbon atom (C2) with the CF_3_ group attached. The Gd−C3′ distance is 2.27 Å in Gd@C_74_(CF_3_) (II), which is slightly longer than the Gd−C1′ distance of 2.20 Å in Gd@C_74_(CF_3_) (I).

The molecular arrangement of the Gd@C_74_(CF_3_)_3_ crystal is rather different from that of the Gd@C_74_(CF_3_) (I) and (II) crystals (figure 2*c*). The triclinic crystal consists of Gd@C_74_(CF_3_)_3_ and Ni(OEP) in a ratio of 2:3 without any solvent molecules. The disordered Gd@C_74_(CF_3_)_3_ molecule was modelled by a Gd atom occupying three independent positions and a C_74_(CF_3_)_3_ molecule with two independent orientations (electronic supplementary material, figure S13). Site occupancies for the major Gd position and the major C_74_(CF_3_)_3_ orientation are 0.63 and 0.74, respectively. Figure 2*f* shows a feasible molecular structure of Gd@C_74_(CF_3_)_3_ consisting of the Gd atom at the major position and the C_74_(CF_3_)_3_ with the major orientation. Three CF_3_ groups of Gd@C_74_(CF_3_)_3_ are attached to carbon atoms on three pentagons of the *D*_3h_-symmetry C_74_ cage. One of the three carbon atoms with CF_3_ groups attached (C2) is the same as the carbon atom with the CF_3_ group attached in Gd@C_74_(CF_3_) (II) shown in figure 2*e*. The Gd position in Gd@C_74_(CF_3_)_3_ is also similar to that in Gd@C_74_(CF_3_) (II). The Gd−C3′ distance is 2.34 Å in Gd@C_74_(CF_3_)_3_, which is slightly longer than that of 2.27 Å in Gd@C_74_(CF_3_) (II).

**Figure 3.**
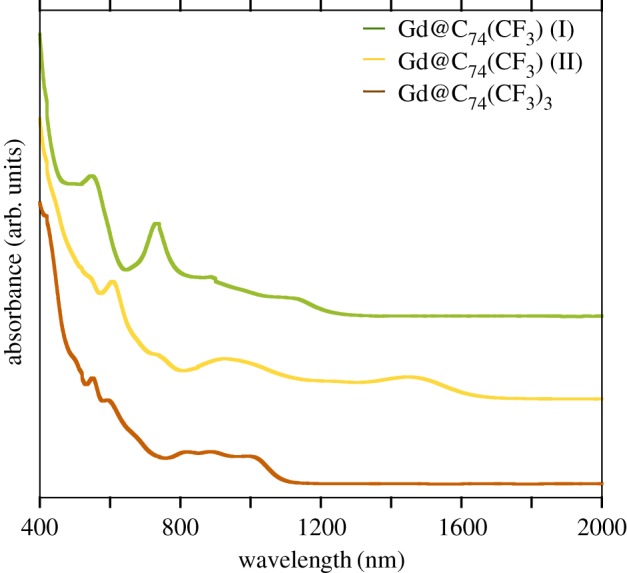
Absorption spectra of Gd@C_74_(CF_3_) (I), Gd@C_74_(CF_3_) (II), and Gd@C_74_(CF_3_)_3_.

Visible–near-infrared (Vis-NIR) spectra of isolated Gd@C_74_(CF_3_) (I and II) and Gd@C_74_(CF_3_)_3_ are shown in figure 3. The quite different spectra of two mono-substituted Gd@C_74_(CF_3_) species show that the two derivatives are isomers having different substituent group positions. HOMO–LUMO gaps of metallofullerenes and their derivatives can be roughly estimated from the onset in absorption spectra. Gd@C_74_(CF_3_) (I), Gd@C_74_(CF_3_) (II) and Gd@C_74_(CF_3_)_3_ have the onset at 1330, 1750 and 1170 nm (figure 2), corresponding to HOMO–LUMO gap of 0.93, 0.71 and 1.06 eV, respectively. The estimated gaps of Gd@C_74_(CF_3_)*_n_* (*n* = 1, 3) are approximately close to HOMO–LUMO gaps of Y@C_74_(CF_3_)*_n_* (*n* = 1, 3), reported in our previous work [16]. In addition, the absorption spectra of Gd@C_74_(CF_3_) (I, II) are also similar to those of two isomers of La@C_74_(C_6_H_3_Cl_2_) [11]. The analogy between these Vis-NIR spectra suggests that the HOMO, LUMO and their neighbouring orbits of M@C_74_ are dominated by features of the cage, irrespective of functional groups as well as encapsulated species.

**Figure 4.**
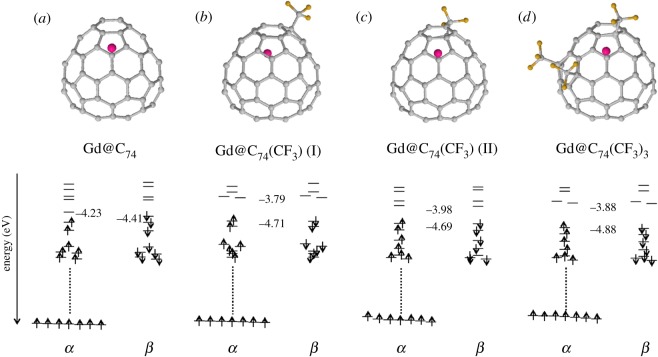
DFT-optimized molecular structures and corresponding calculated molecular orbital energy levels of (*a*) Gd@C_74_, (*b*) Gd@C_74_(CF_3_) (I), (*c*) Gd@C_74_(CF_3_) (II) and (*d*) Gd@C_74_(CF_3_)_3_.

Density functional calculations were performed to obtain more detailed information on the trifluoromethyl derivatives. On the basis of the structures obtained by X-ray crystal structure analysis, we carried out geometry optimizations and energy calculations on isolated pristine and functionalized Gd@C_74_. Aside from small bond length changes attributable to the exchange correlation functional used, the molecular structures from X-ray analysis were confirmed as stable. Figure 4 shows optimized structures and molecular orbital energy levels. Gd@C_74_ is an open-shell system in agreement with the discussion above, with HOMO energy of –4.41 eV and LUMO energy of –4.23 eV, confirming that Gd@C_74_ has a very small HOMO–LUMO gap. The states lie higher in the gap than for the subsequent CF_3_ functionalized species, in agreement with the experimental observation of a prevalence of positively charged species in the mass spectra. Once functionalized with CF_3_ the calculated HOMO–LUMO gaps are significantly increased, with gaps for Gd@C_74_(CF_3_) (I), Gd@C_74_(CF_3_) (II) and Gd@C_74_(CF_3_)_3_ of 0.92, 0.71 and 1.00 eV, respectively, consistent with those estimated from the absorption onsets. It is clear that the energy gap is enlarged considerably upon exohedral trifluoromethylation, and all three species have closed-shell configurations confirming the demonstrated stability of odd-number CF_3_ additions. All three have fully unpaired Gd *f*-states, which are a pre-requisite for biomedical applications such as MRI contrast agents [3–6].

## 4. Discussion

The present trifluoromethylation method provides only mono- and tri-substituted derivatives [16–18]. One of the possible reasons for the selectivity is that it has an open-shell structure. Valence electrons are transferred from Gd to the cage, resulting in a charge distribution of Gd^3+^@C_74_^3−^ [10]. Addition of odd-number CF_3_ groups to the carbon cage may result in a closed-shell configuration, which is more stable than an open-shell configuration of non-substituted Gd@C_74_. A similar trend has been observed in the case of previous yttrium- and lanthan-encapsulated metallofullerenes [16].

It is interesting also to examine the binding energy of CF_3_ groups to the Gd@C_74_ cage. Considering the reaction

$$n\frac{1}{2}{(C{\text{F}}_{3})}_{2}+\text{Gd}@{\text{C}}_{74}\to \text{Gd}@{\text{C}}_{74}{\left(\text{C}{\text{F}}_{3}\right)}_{n},$$

we obtain a CF_3_ binding energy for the two *n* = 1 isomers of 6.26 and 7.73 kcal mol^−1^, respectively. These are close to each other, explaining the presence of both isomers (i.e. there is no single CF_3_ functionalized isomer with significantly higher thermodynamic stability than any other). At the same time, the binding energy is considerably lower than for Gd@C_60_ [19], which averages at 10.35 kcal mol^−1^ per CF_3_ group for Gd@C_60_(CF_3_)_5_. In the case of the Gd@C_74_ *n* = 3 isomer the average binding energy per CF_3_ groups drops even further to 5.78 kcal mol^−1^ (i.e. the two new CF_3_ groups are each bound by only 4.81 kcal mol^−1^), and it is reasonable to assume from this that subsequent pairwise CF_3_ addition will be even less stable, presumably explaining the absence of stable *n* = 5 addition species.

## 5. Conclusion

In conclusion, the fullerene derivatives Gd@C_74_(CF_3_) (I), Gd@C_74_(CF_3_) (II) and Gd@C_74_(CF_3_)_3_ were isolated by *in situ* trifluoromethylation followed by multi-stage HPLC purification. Their chemical structures were determined by X-ray crystal structure analysis and all of Gd@C_74_ isomers have *D*_3*h*_-symmetry cages. CF_3_ groups are attached to carbon atoms on pentagons of fullerene cages. Formation of closed-shell systems and HOMO–LUMO gap widening of Gd@C_74_ by the one- or three-fold addition of CF_3_ group were confirmed by Vis-NIR spectra measurement and computational modelling.

## Supplementary Material

Supporting Figures

## Supplementary Material

C74

## Supplementary Material

Gd@C74

## Supplementary Material

Gd@C74(CF3)-1

## Supplementary Material

Gd@C74(CF3)-2

## Supplementary Material

Gd@C74(CF3)3

## Data accessibility

The crystal structures are freely available from the Cambridge Crystallographic Data Centre (CCDC) with CIF numbers 1824999 for Gd@C_74_(CF_3_) (I), 1825000 for Gd@C_74_(CF_3_) (II) and 1825001 for Gd@C_74_(CF_3_)_3_. All DFT calculated structures (in xyz format) discussed in this article are provided in the electronic supplementary material associated with this article.
