# Supplementary material for: Isolation and structure determination of missing fullerenes Gd@C74(CF3)n through in situ trifluoromethylation
Source: R Soc Open Sci. 2018 Sep 19;5(9):181015. doi: 10.1098/rsos.181015 (PMC6170568; doi:10.1098/rsos.181015)
Supplement: Supporting Figures [file rsos181015supp1.doc]

**Isolation and Structure Determination of Missing Fullerenes Gd@C_74_(CF_3_)*_n_* through *in-situ* Trifluoromethylation**

**Ayano Nakagawa,^1^ Shinobu Aoyagi,*^2^ Haruka Omachi,*^1,3^ Katsuma Ishino,^1^ Makiko Nishino,^1^ Jeremy Rio,^4^ Chris Ewels,*^4^ and Hisanori Shinohara^1,5^**

*^1^ Department of Chemistry, Graduate School of Science, Nagoya University, Nagoya, 464-8602, Japan*

*^2^ Department of Information and Basic Science, Nagoya City University, Nagoya, 467-8501, Japan*

*^3^ Research Center for Materials Science, Nagoya University, Nagoya, 464-8602, Japan*

*^4^ Institut des Materiaux Jean Rouxel (IMN), Université de Nantes, CNRS UMR6502, 2 Rue de la Houssiniere, BP32229, Nantes, 44322, France*

*^5^ Institute for Advanced Research, Nagoya University, Nagoya, 464-8602, Japan*

**Figure S1:** A cross-sectional view of the DC arc-discharge apparatus for the synthesis of Gd- metallofullerenes. Polytetrafluoroethene (PTFE) rods are placed near the discharge area for the *in situ* CF_3_-functionalization.


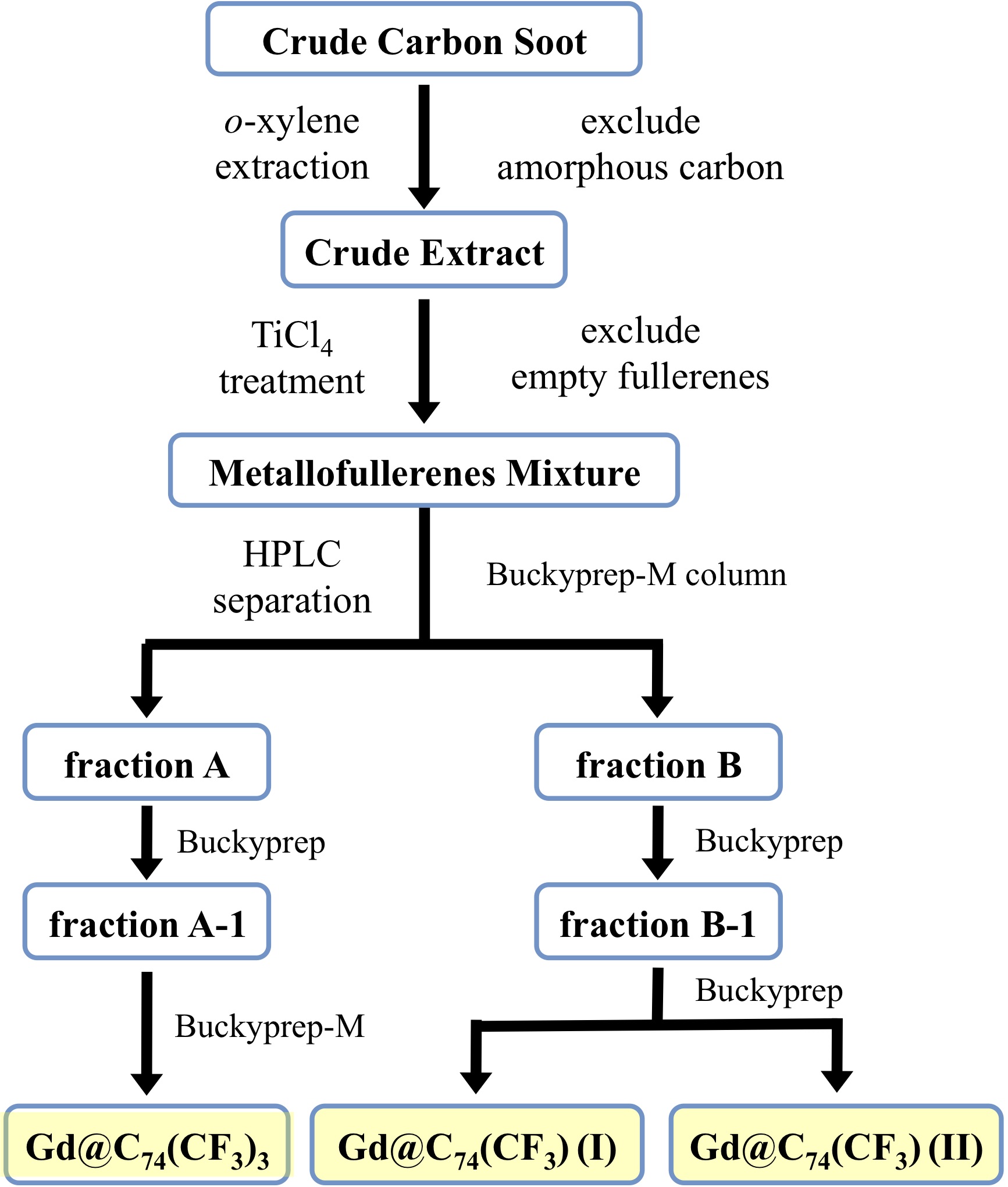


**Figure S2:** The overall separation and isolation scheme of Gd@C_74_(CF_3_) (I), -(II), and Gd@C_74_(CF_3_)_3_ from raw soot.

**Figure S3:** The first stage HPLC profile (Buckyprep-M column; flow rate: 21 mL/min). The metallofullerene Gd@C_74_(CF_3_)_3_ was contained in fraction A (6.5-9 min). Two isomers of Gd@C_74_(CF_3_) were contained in fraction B (10-11 min).

**Figure S4:** The second stage HPLC profile of fraction A (Buckyprep column; flow rate: 16 mL/min). The fractions A-1 contain Gd@C_74_(CF_3_).

**Figure S5:** The third stage HPLC profile of fraction A-1 (two Buckyprep-M columns connected in series; flow rate: 12 mL/min). Gd@C_74_(CF_3_)_3_ was contained in the fraction A-1-1.

**Figure S6:** The second stage HPLC profile of fraction B (Buckyprep column; flow rate: 16 mL/min). The fractions B-1 and B-2 contains Gd@C_74_(CF_3_)

**Figure S7:** The third stage HPLC profile of mixture fraction B-1 and B-2 (Buckyprep column; flow rate: 16 mL/min). The first peak is Gd@C_74_(CF_3_) (I) and the second peak is Gd@C_74_(CF_3_) (II).


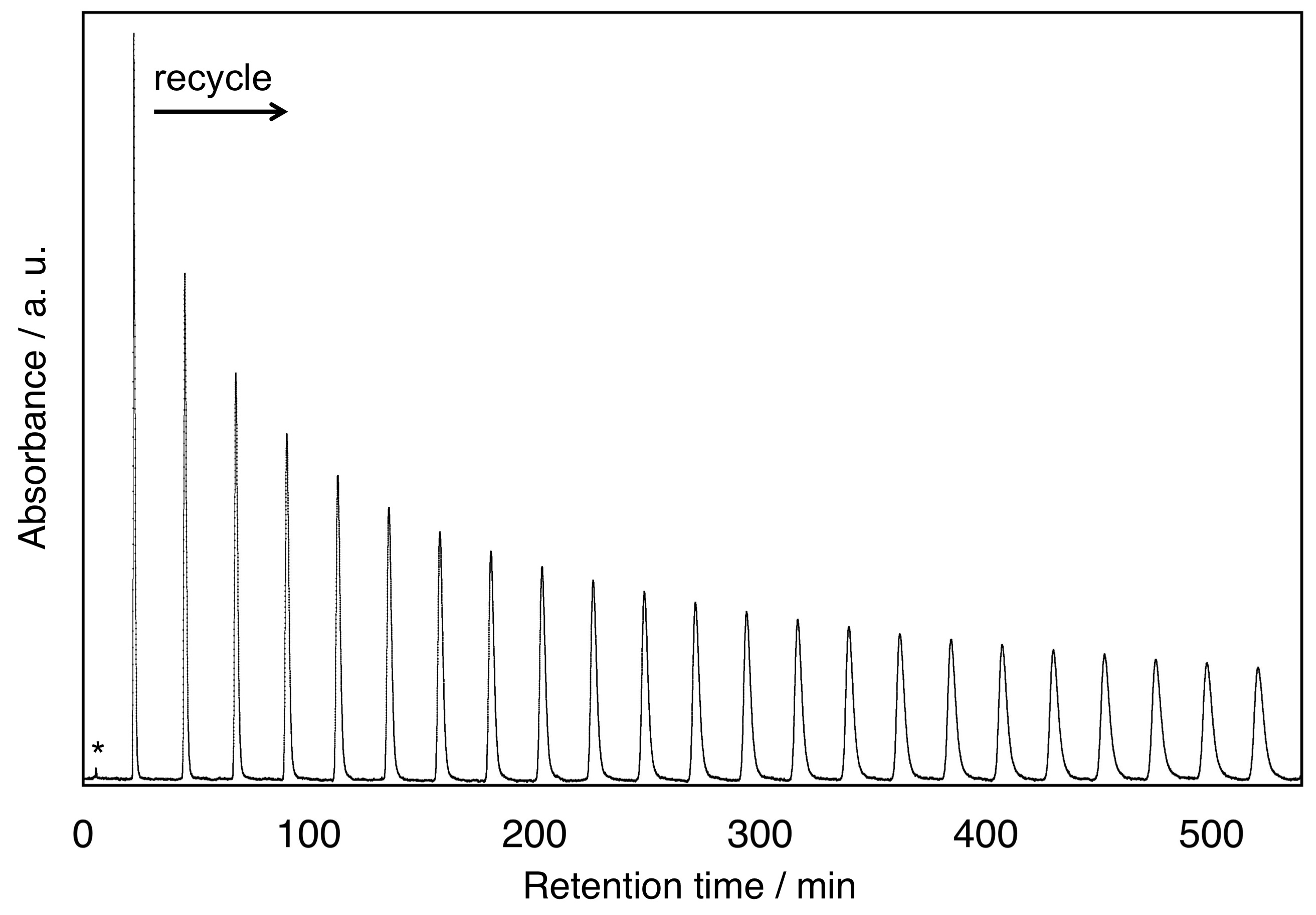


**Figure S8:** The recycled HPLC profile of purified Gd@C_74_(CF_3_) (I) (Buckyprep column; flow rate: 16 mL/min). Asterisk (*) peak is for residual CS_2_.


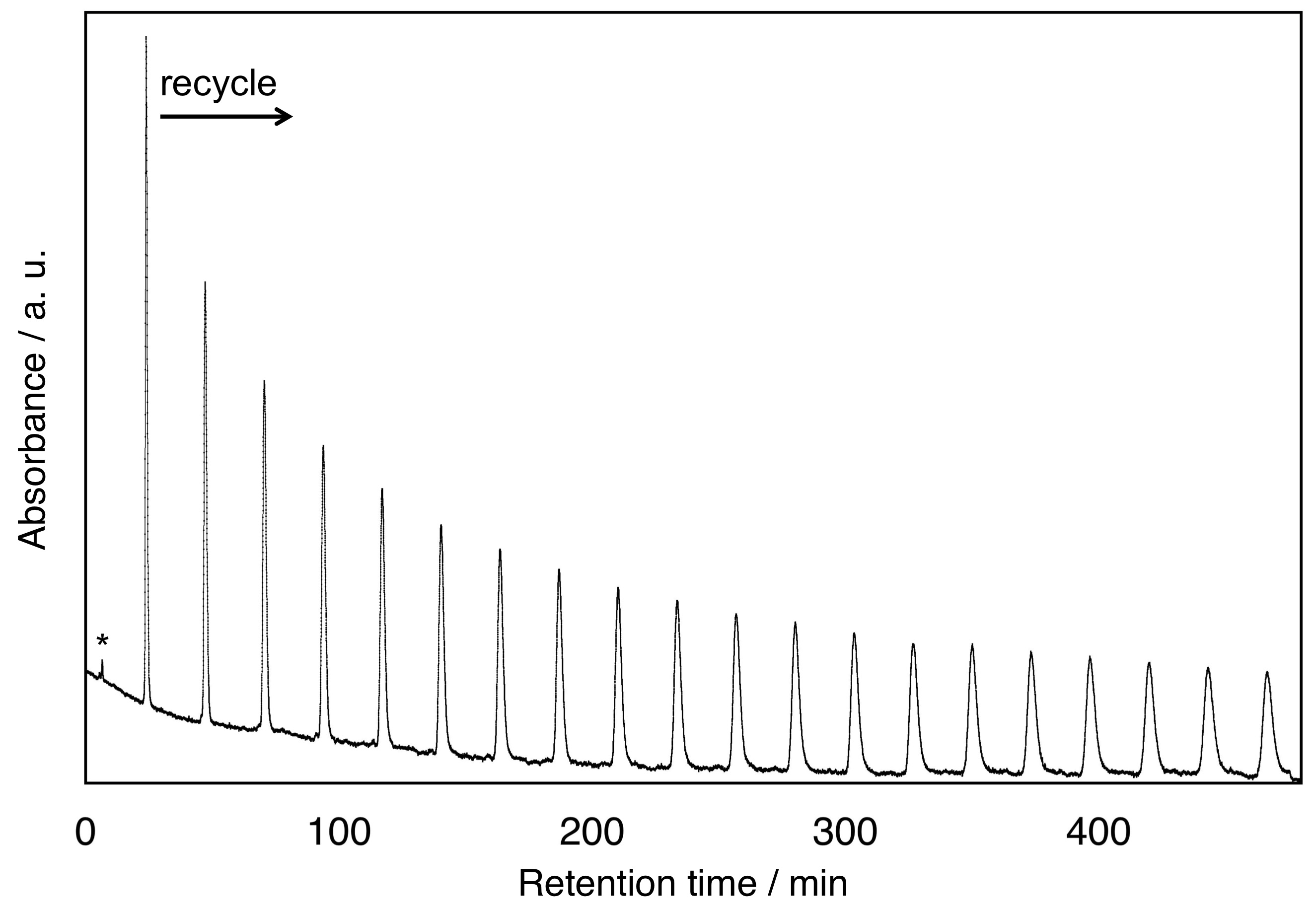


**Figure S9:** The recycled HPLC profile of purified Gd@C_74_(CF_3_) (II) (Buckyprep column; flow rate: 16 mL/min). Asterisk (*) peak is for residual CS_2_.


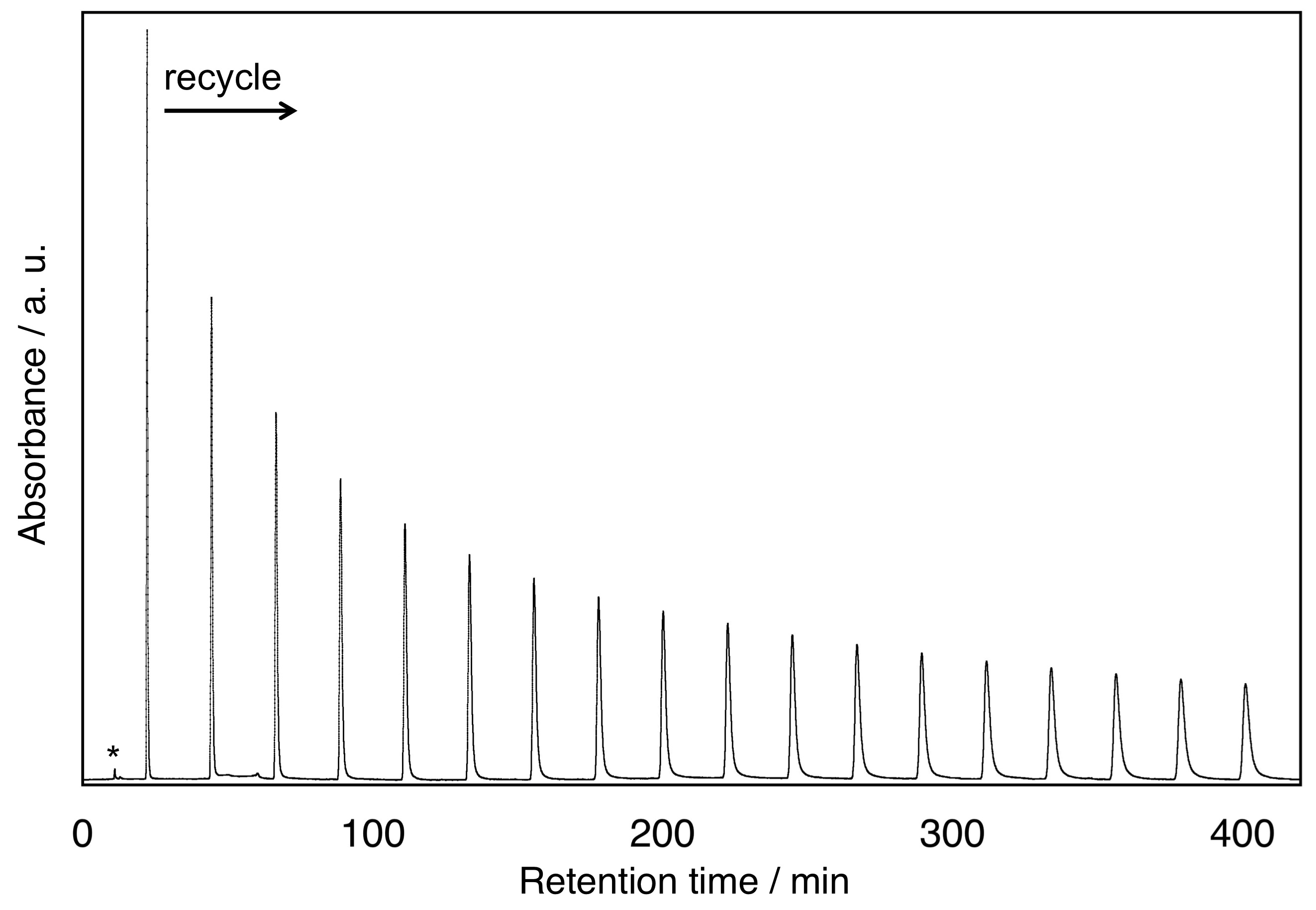


**Figure S10:** The recycled HPLC profile of purified Gd@C_74_(CF_3_)_3_ (two Buckyprep-M columns connected in series; flow rate: 12 mL/min). Asterisk (*) peak is for residual CS_2_.

**Table S1**. Crystallographic data for Gd@C_74_(CF_3_) (I), -(II) and Gd@C_74_(CF_3_)_3_ co-crystallized with Ni(OEP) from solutions.

|  | Gd@C_74_(CF_3_) (I) | Gd@C_74_(CF_3_) (II) | Gd@C_74_(CF_3_)_3_ |
| --- | --- | --- | --- |
| formula | GdC_75_F_3_∙NiC_36_N_4_H_44_∙  CHCl_3_∙0.50C_7_H_8_ | GdC_75_F_3_∙NiC_36_N_4_H_44_∙  0.17CS_2_∙0.47C_7_H_8_ | 2GdC_77_F_9_∙3NiC_36_N_4_H_44_ |
| formula weight | 1871.89 | 1762.31 | 4280.42 |
| crystal size (mm) | 0.05 × 0.05 × 0.01 | 0.05 × 0.05 × 0.01 | 0.10 × 0.05 × 0.01 |
| temperature (K) | 100 | 100 | 100 |
| X-ray wavelength (Å) | 0.4959 | 0.7022 | 0.6227 |
| crystal system | monoclinic | monoclinic | triclinic |
| space group | *C*2/*m* | *C*2/*m* | *P*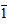 |
| unit cell parameters | *a* = 24.7330(5) Å  *b* = 15.458(2) Å  *c* = 18.8412(7) Å  *β* = 92.042(2)°  *V* = 7198.8(10) Å^3^ | *a* = 24.561(2) Å  *b* = 15.2677(13) Å  *c* = 18.5789(17) Å  *β* = 91.968(6)°  *V* = 6962.9(11) Å^3^ | *a* = 15.144(5) Å  *b* = 15.193(5) Å  *c* = 18.595(6) Å  *α* = 87.940(6)°  *β* = 88.638(6)°  *γ* = 86.146(6)°  *V* = 4265(2) Å^3^ |
| *Z* | 4 | 4 | 1 |
| No. of independent reflections | 10,353 (*d* > 0.70 Å) | 7,205 (*d* > 0.80 Å) | 11,737 (*d* > 0.80 Å) |
| Σ*σ_I_*/Σ*I* | 0.0576 | 0.0854 | 0.2283 |
| No. of parameters | 324 | 303 | 1163 |
| *R*1 | 0.0926 (|*F*| > 4*σ*) | 0.1096 (|*F*| > 4*σ*) | 0.1497 (|*F*| > 4*σ*) |
| *wR* | 0.2412 (|*F*| > 4*σ*) | 0.2846 (|*F*| > 4*σ*) | 0.3113 (|*F*| > 4*σ*) |
| GOF | 1.464 (|*F*| > 4*σ*) | 1.728 (|*F*| > 4*σ*) | 1.291 (|*F*| > 4*σ*) |
| CIF deposition No. | 1824999 | 1825000 | 1825001 |


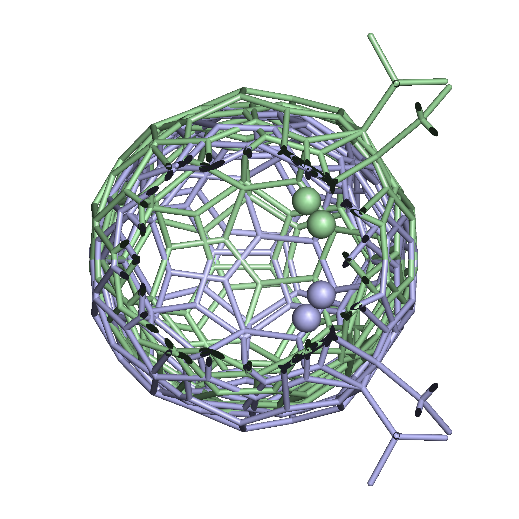


**Figure S11:** Disordered structure of Gd@C_74_(CF_3_) (I). Green and blue parts are equivalent by the mirror symmetry. Site occupancies for the two independent Gd positions are 0.64 and 0.36, respectively. Site occupancies for the three independent C_74_(CF_3_) (I) orientations are 0.36, 0.39, and 0.25, respectively.


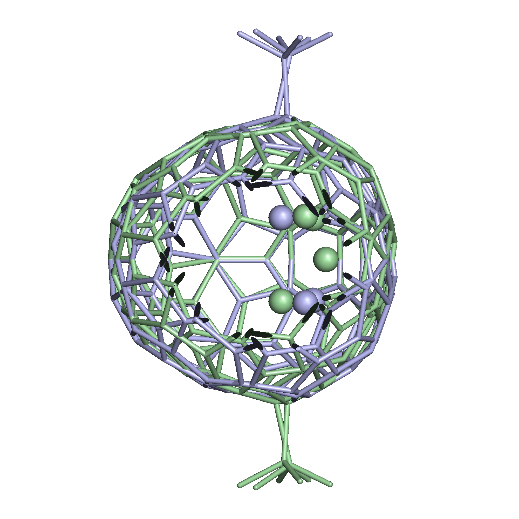


**Figure S12:** Disordered structure of Gd@C_74_(CF_3_) (II). Green and blue parts are equivalent by the mirror symmetry. Site occupancies for the four independent Gd positions are 0.48, 0.29, 0.16, and 0.07, respectively. Site occupancies for the two independent C_74_(CF_3_) (II) orientations are 0.61 and 0.39, respectively.


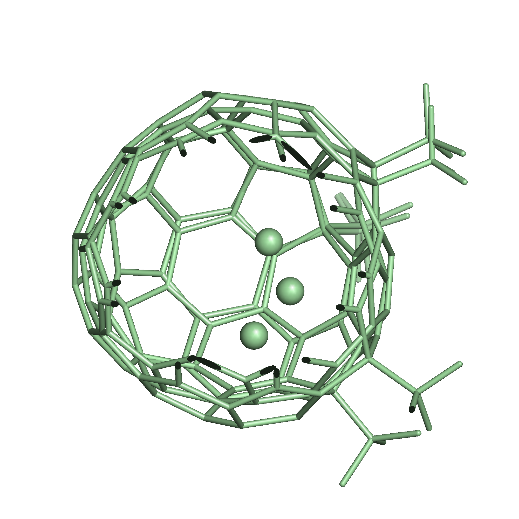


**Figure S13:** Disordered structure of Gd@C_74_(CF_3_)_3_. Site occupancies for the three independent Gd positions are 0.63, 0.24, and 0.13, respectively. Site occupancies for the two independent C_74_(CF_3_)_3_ orientations are 0.74 and 0.26, respectively.
